# An online survey and interview of GPs in the UK for assessing their satisfaction regarding the medical training curriculum and NICE guidelines for the management of menopause

**DOI:** 10.1177/20533691221106011

**Published:** 2022-05-31

**Authors:** Nitya Dintakurti, Shreya Kalyanasundaram, Prashant Jha, Vikram Talaulikar

**Affiliations:** 1 RU Medical Ltd., London, UK; 2 Department of Biomedical Engineering and Imaging Sciences, 4616 King’s College London, London, UK; 3 Department of Reproductive Medicine, 8964 UCLH, London, UK

**Keywords:** Menopause, education, general practitioners, management, postmenopause, perimenopause

## Abstract

**Background:**

The UK is home to 13 million menopausal women. The aim of this study was to determine the views of GPs on their levels of confidence and comfort when advising or treating menopausal women and assess the need for further training.

**Method:**

An anonymous online questionnaire was sent out to GPs working within the NHS across the UK between January 2021 and March 2021. The questionnaire was circulated via GP e-mail lists, Facebook, and LinkedIn, and included an option for respondents to volunteer for a semi-structured interview.

**Results:**

The questionnaire had 173 responses. 52% of GPs indicated that they were not offered enough support to be able to advise and treat women with menopausal symptoms appropriately. 77.5% of GPs expressed that there is a need to improve training provided on menopause in medical school and GP training. 60.7% of GPs felt comfortable managing menopausal women and offering them management options. 22.5% of the respondents felt that the NICE guidelines are easy and clear guidance for clinical practice. Five GPs were further interviewed, and the analysis of the responses showed the perceived need by the GPs for improvements in medical training regarding menopause.

**Conclusion:**

There is a need for better support and medical training for GPs to help them advice and treat women with menopausal symptoms. This is key for ensuring that every woman in the UK feels supported in their journey during the menopausal transition and is offered evidence-based advice to help them make informed decisions.

## Introduction

Menopause is the permanent cessation of menstrual cycles for 12 months or more resulting from declining numbers of follicles and estrogen production from the ovaries and is not associated with a pathology.^1,2^ Every woman’s experience of menopausal transition is unique and more than 30 different symptoms have been attributed to menopause. Some of the most common symptoms include hot flashes, night sweats, vaginal dryness, decreased libido, insomnia, fatigue, mood changes sleep difficulties, brain fog, and joint pain.^3^ There are currently 13 million menopausal women in the UK out of which it is estimated that there are around 4.3 million women aged 50 and over in employment.^4^ This number is only growing every year meaning an increased number of working women will experience menopause during their employment.

General practitioners or GPs are generally the first point of contact between a clinician and a woman facing menopausal symptoms.^5^ This makes the GP training of utmost importance as it must equip GPs with the relevant tools to manage and treat menopausal symptoms. Women make up a huge part of the workforce, and there has been a steep increase in employment rates over the last 30 years for women aged 60–64 (from 18% to 41%) and for women aged 55–59 (from 49% to 69%). Access to health professionals who are up to date with evidence-based menopause care is key to make sure that these women receive the help if and when they need it as menopausal symptoms can deteriorate the quality of work, increase absence days and overall impact the UK economy hugely.

The British Menopause Society (BMS) is a non-profit organization established in 1989 which aims to educate, inform, and guide Healthcare professionals on menopause and all aspects of post reproductive health via lectures, conferences, meetings, and exhibitions. The number of BMS recognized menopause specialists in the country remains small and too many women are unable to access menopause care or support suggesting there is an urgent need for more evidence-based menopause care to occur in the UK. We conducted this survey study to understand the views and perceptions of GPs in the UK about menopause training during undergraduate and GP training years and the support available to them when offering menopause care.

## Methods

The inclusion criteria for this study were all GPs working within the NHS across the UK. The study included filling in an anonymous online questionnaire and further volunteering to take part in semi-structured interviews which were designed to assess their confidence level when treating women presenting with menopausal symptoms and whether they believed that they received adequate training during their medical education to manage menopause related health issues.

The online questionnaire was circulated via GP e-mail lists on the NHS “Find a GP” service and groups on social media networks like Facebook and LinkedIn. The questionnaire also collected the e-mail addresses of GPs who volunteered to provide contact information and further take part in a semi-structured interview. Interviews continued until data saturation was reached, when those analyzing the data agreed no new themes were being identified from further interviews. The questions were framed as tests of opinion on a Likert Scale ranging from “Strongly Agree” to “Strongly Disagree.”

The questions were:

1. In my opinion, my GP training offered me enough support and the necessary tools to be able to advise and treat women with menopausal symptoms appropriately.
2. I find the NICE menopause care guidelines clear and easy to practice (https://www.nice.org.uk/guidance/ng23/chapter/Recommendations)
3. As a GP, I feel comfortable managing patients with health problems related to menopause and offering them HRT or non-HRT treatment options.
4. In my opinion, there is a need to improve training provided on menopause and related health issues in the undergraduate curriculum and during GP training.

The semi-structured interview questions addressed the most common symptoms women experienced, the options given to women to manage the menopause, compliance for HRT, management options provided for women with comorbidities, awareness about menopause-related medical technologies, adequacy of medical training about menopause, and suggestions to make it easier for GPs to treat women with menopausal issues. (Refer to Appendix 1 for full list of questions.)

We are aware that the study design has an intrinsic bias. It is likely that the respondents would be GPs with an interest in menopause.

## Results

A total of 173 GPs responded to the online questionnaire and a further 22 provided e-mail addresses for supporting further research. Of these, five responded favorably to participate in semi-structured, qualitative virtual interviews.

**Figure 1.**
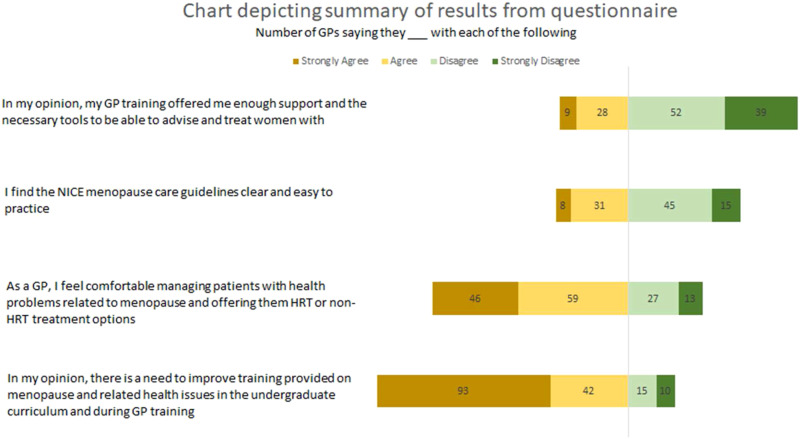
Chart depicting summary of results from online questionnaire.

The combined results are summarized in Table 1 and Figure 1.

**Table 1.** Summary of results from questionnaire.

Questions	Strongly agree	Agree	Neutral	Disagree	Strongly disagree
GP training offered enough support and the necessary tools to be able to advise and treat women with menopausal symptoms appropriately	9 (5.2%)	28 (16.2%)	46 (26.6%)	51 (29.5%)	39 (22.5%)
The NICE menopause care guidelines are clear and easy to practice	8 (4.6%)	31 (17.9%)	75 (43.4%)	44 (25.4%)	15 (8.7%)
I feel comfortable managing patients with health problems related to menopause and offering them HRT or non-HRT treatment options	46 (26.6%)	59 (34.1%)	28 (16.2%)	27 (15.6%)	13 (7.5%)
There is a need to improve training provided on menopause and related health issues in the undergraduate curriculum and during GP training	93 (53.8%)	41 (23.7%)	14 (8.1%)	15 (8.7%)	10 (5.8%)

52% of respondents disagreed with their GP training equipping them with the right tools and support to treat and manage women presenting with menopausal symptoms and 26.6% of the respondents neither agreed nor disagreed to this. This statistic is concerning as more than half of the respondents felt a lack of adequate training and support for menopause care in their GP practice. All the GPs we interviewed needed to seek out extra resources like e-learning modules, menopause related books, courses from the BMS and the Royal College of General Practitioners (RCGP), and journal articles to treat menopausal symptoms confidently. This possibly indicates that there may be a number of GPs without a special interest in menopause who may not seek out these additional resources making them less equipped to handle menopausal symptoms confidently.

The results of the survey showed a unanimous (77.5%) need to improve training provided on menopause and related health issues in the undergraduate curriculum and during GP training. The results of the question “As a GP, I feel comfortable managing patients with health problems related to menopause and offering them HRT or non-HRT treatment options” showed that 60.7% of respondents agreed with the statement. Even though a large majority of the respondents agreed that there is a need to improve GP training, more than half the respondents do feel confident in treating women presenting with menopausal symptoms. This again possibly indicates the extra effort GPs have been putting into getting up to date with the latest evidence-based information about menopause care so that they are confident in their practice.

Only 22.5% of the respondents felt that the NICE guidelines are easy and clear guidance for clinical practice. This again suggests that the GPs have considerable difficulty applying these guidelines in practice and is likely to be linked to the lack of confidence faced by GPs when treating menopausal women.

The semi-structured interviews conducted lasted approximately 20 min and helped gain insight into the extent of lack of training with regards to menopause. Every interview revealed the severe need for more support and GP training. Some of the statements from the GPs that were interviewed which captured the essence of the conversations are:

*“No training with regards to menopause at all. Don’t remember being taught about menopause in medical school.”*

*“Training programs are mostly taught by males. Depending on who is overseeing the training and who is running training sessions, menopause training varies from person to person. The training provided was enough to get by exam but not ideally.”*

*“Majority of the GPs roll their eyes about HRT.”*

*“Some doctors here are not comfortable with HRT either.”*

*“There wasn’t any information about menopause but has become better in more recent years.”*

It was observed that many GPs had not received any formal menopause training during their GP education. Considering the high prevalence, interdisciplinary involvement, and complexity of menopausal health issues, it is important that the GPs receive appropriate training to manage and treat menopause.

We also discovered that it is up to the GPs to find and voluntarily enroll for menopause-related CME (continuing medical education) from providers like the RCGP, British menopause Society, among others.

## Discussion

Doctor’s appointments can be stressful experiences and even more so during the menopause when women are feeling anxious, depressed, forgetful, and generally overwhelmed by their symptoms. This data indicates that there is a strong need for better medical education and training about the menopause for GPs who are the first line of medical consultations in the UK, even though this natural phase in a woman’s life cycle will affect possibly half of their patients directly and almost all their patients indirectly. There are 54,024 GPs in England & Scotland and according to the British Menopause Society only 62 GPs of them are recognized menopause specialists in the UK (United Kingdom).^6^ This means that while the GPs are the main source of advice for the majority of patients seeking menopause care not all of them feel that they have had a structured training in menopausal care.

Some studies have indicated that the flawed findings of the 2002 Women’s Health Initiative study are still being used by some medical practitioners as the basis for their advice to patients even though there have been more current studies and re-analysis of existing data with contrasting results.^7^

Interestingly, even though most GPs said that they were not offered enough support and the necessary tools to be able to advise and treat women with menopausal symptoms appropriately, most of them still said that they were comfortable managing patients with health problems related to menopause and offering them HRT or non-HRT treatment options. This possibly suggests that GPs have had to take an initiative to better educate themselves about menopause and using additional resources like books, online courses, etc. to help them advice and treat women as per latest evidence-based guidelines.

There is a need for effective strategies to provide better support and menopause related training for GPs. This could potentially take the form of mandatory courses in menopause offered by the Royal College of General Practitioners (RCGP)**.** In May 2021, RCGP announced via a press release that menopause training is included in the GP training curriculum and mentioned “GPs have the broadest curriculum, yet shortest training programme of any medical specialty, which aims to expose trainees to the full breadth of conditions they are likely to see in general practice. Introducing additional mandatory training courses for some areas of medicine and not others would be unworkable.” The press release further reinforces our findings and this education gap.^8^ The British Menopause Society currently runs training courses for GPs, but these are optional, and clinicians are under no obligation to attend them. We wonder if the some of the CPD (continuing professional development) hours could be mandatorily fixed for menopause education.

As a summary, our results indicate the need of a wider and deeper education campaign for GPs to improve menopause care in the UK.

## Conclusion

Around half the world’s adult population is going to experience menopause. Modern medicine has enabled the improvement of a woman’s life expectancy, but there’s little joy in living longer if women are not healthy as they age.

Our study, although limited in scope by the chance only those GPs with an interest in menopause would have participated, suggests the need for better support and medical training in menopause for GPs in the UK. This will help both, the women needing the right advice from their GPs and the GPs wanting to support women in their journey of menopausal transition.
